# Exploring the relationships among music performance anxiety, teaching anxiety, and self-efficacy of Chinese preservice music teachers

**DOI:** 10.3389/fpsyg.2024.1373454

**Published:** 2024-04-12

**Authors:** Cancan Cui, Xin Xie, Yue Yin

**Affiliations:** ^1^College of Music and Dance, Guangzhou University, Guangzhou, Guangdong, China; ^2^School of Music, The Pennsylvania State University, State College, PA, United States; ^3^Allegheny Singer Research Institute, Allegheny Health Network, Pittsburgh, PA, United States

**Keywords:** teaching anxiety, music performance anxiety, self-efficacy, relationships, preservice music teachers

## Abstract

This quantitative study aimed to explore the relationships among music performance anxiety (MPA), teacher anxiety (TA), and self-efficacy (SE) through a survey study of Chinese preservice music teachers (*N* = 237). We also examined gender, grade, primary instrument, secondary instrument, music learning time length, and time spent in four activities: peer teaching, practicum, internship, and private teaching as potential predictors of MPA, TA, and SE. Results indicated that the higher the self-efficacy, the lower music performance anxiety and teaching anxiety; Simultaneously, the increased music performance anxiety was associated with an increased teaching anxiety. Partial correlation results indicated a significant but negative correlation between TA and SE with MPA controlled. Teaching anxiety, followed by primary piano, was the strongest predictor of MPA. MPA, followed by SE and peer teaching, was the strongest predictor of TA. TA, followed by grade level, was the strongest predictor of SE. The results from the multivariate analysis of variance revealed that the SE of male preservice music teachers were significantly higher than their female counterparts. As a study implication, music teacher educators may consider interventions and support mechanisms that address both types of anxiety simultaneously to improve overall teacher preparedness.

## Introduction

The role of a music teacher is challenging and demanding. A qualified music teacher must possess various types of abilities, such as (a) administration, (b) classroom management, (c) musicianship, and (d) content and pedagogical knowledge (Hourigan and Scheib, 2009). As required by the Ministry of Education, People’s Republic of China, (2021), teacher candidates must teach ethically, comprehensively, independently, and practically (Ministry of Education, People’s Republic of China, 2021). Chinese music scholars have further elaborated on the teaching practice abilities needed of music teachers, encompassing two aspects: musicianship and pedagogy (Chen, 2016). These official standards serve as a testament to the critical importance of music teachers’ ability to demonstrate their musicianship while teaching effectively.

In essence, music teachers assume dual roles, facing the duality between the concepts of “musicians” and “teachers.” Extensive research has consistently highlighted the challenges faced by music teachers, particularly preservice music teachers, who encounter a significant discrepancy between their expectations and the reality of their teaching and musical modeling (Ballantyne, 2007; Strong, 2013). This incongruity often leads to increased levels of anxiety, which, in turn, could impact their self-efficacy. It is noteworthy that previous studies have predominantly focused on preservice music teachers’ performance anxiety and self-efficacy beliefs individually (Hargreaves et al., 2007; Egilmez, 2015). The complexity of music performance anxiety is closely linked with other forms of anxiety which are contextual, such as social anxiety and test anxiety (Papageorgi et al., 2011). Our study assumed an interconnectedness between performance anxiety and teaching anxiety in the music classrooms, with their relationship potentially influenced by individual self-efficacy levels.

## Key concepts

Parallel to the empirical investigation of music performance anxiety (MPA), self-efficacy (SE), and teaching anxiety (TA) as separate subjects, the underlying concepts have been developed separately. Specifically, we reviewed these three concepts and further examined and discussed the related literature.

### Music performance anxiety

Music Performance Anxiety (MPA) refers to “the fear that grips individuals before or during a performance and is often likened to ‘stage fright’ or a fundamental fear of life itself” (Goode, 2004, p. 25). Research has indicated that several factors contribute to musicians’ performance anxiety, including gender (Patston and Osborne, 2016), age (Dempsey and Comeau, 2019), individual characteristics (Smith and Rickard, 2004), personality traits (Girgin, 2017), anxiety-related traits (Hallam and Welsh, 2007), early childhood relationships (Kenny, 2009), psychological vulnerability (Kenny, 2009, 2011), and proximal performance vulnerability (Kenny, 2009, 2011). Among these, factors such as low self-esteem, low self-efficacy, inadequate preparation, a surface approach to learning, and high task difficulty and values have negative effects on an individual’s experience of music performance anxiety. However, some researchers argue that a certain level of tension before an event is natural and may enhance the experience; in this context, music performance anxiety might be seen as a potential benefit for music students and musicians (Kokotsaki and Davidson, 2003).

To date, music teachers, undergraduate music majors, and professional musicians continue to grapple with various physiological, psychological, emotional, and behavioral challenges when performing in front of others (Ely, 1991; Kenny, 2009, 2011; Papageorgi et al., 2011; Papageorgi and Welch, 2020; Papageorgi, 2021). Preservice music teachers, who are in the process of gaining experience in teaching and performing in front of students, may also be susceptible to the effects of music performance anxiety (Taborsky, 2007).

### Teacher self-efficacy

Self-efficacy (SE), a component of self-concept, refers to the perceived belief in one’s capabilities to organize and execute actions necessary for achieving specific goals (Bandura, 1997, p. 3). Teacher self-efficacy has a potential impact on teachers’ behaviors, and consequently, students’ behaviors, it has strong influence on teaching performance, instructional effectiveness, and student outcomes (Bandura, 1997; Klassen and Tze, 2014).

Due to the multidimensional nature of teacher self-efficacy (Biasutti et al., 2021), there are positive correlations between teacher self-efficacy and variables such as teaching experience (Tschannen-Moran and Hoy, 2007; Potter, 2021), school context (urban, suburban, rural/small town) (Tschannen-Moran and Hoy, 2007), and contextual familiarity during field placements (Regier, 2021). Positive associations have also been noted between teacher self-efficacy and various aspects, including students’ academic adjustment, teacher behavior, and practices related to classroom quality (Zee and Koomen, 2016).

Self-efficacy was found malleable (Bandura, 1997); preservice music teachers’ self-efficacy can be formed by curricular experiences. Prichard (2017) found that teaching experiences, such as individualized mentoring, peer teaching, and structured field observations, positively influenced the pre-service music teachers’ efficacious beliefs. Bergee (2002) found that preservice music teachers’ self-efficacy for classroom management improved after viewing video recordings or applying learned classroom management strategies in the field. In Potter’s (2021) study, pre-service teachers’ self-efficacy was found to be impacted by teaching experience. Results further indicated that gains of the field application group lasted longer than those of the video-only group-a result that supports Bandura’s (1997) assertion that mastery experiences are often most impactful on efficacious beliefs.

### Teaching anxiety

Teaching anxiety (TA), as defined by Gardner and Leak (1994), relates to the anxiety experienced in connection with teaching activities, especially those involving the preparation and execution of classroom activities. In the realm of mathematics education, research has revealed that the content knowledge dimension of mathematics teaching anxiety can have a detrimental impact on various aspects of self-efficacy beliefs related to mathematics teaching. These aspects encompass teaching efficacy, motivation, taking responsibility, and teaching effectiveness. Additionally, studies have indicated that the teaching knowledge dimension of mathematics teaching anxiety negatively affected the dimension of effective teaching (Peker, 2016). In a related study, Olson and Stoehr (2019) made an important discovery, highlighting that math anxiety and math teaching anxiety are not confined solely to evaluative contexts. Instead, when anxiety is triggered by thoughts of evaluation, preservice teachers may experience concerns not only about their own performance but also about the performance of their students. However, teaching anxiety is commonly observed in the general education field (Patkin and Greenstein, 2020; Liu et al., 2022), there is limited literature addressing teaching anxiety in the field of music education.

## Purpose and research questions

Previous studies concentrated on the relationship between music performance anxiety and self-efficacy in musicians and music teachers (Hargreaves et al., 2007; Egilmez, 2015; Girgin, 2017; Dempsey and Comeau, 2019; MacAfee and Comeau, 2020). A few studies have explored teaching anxiety in relation to teaching other subjects (Peker, 2009), such as math education (Patkin and Greenstein, 2020) and linguistic education (Liu et al., 2022). As far as can be determined, no existing study has placed a specific focus on examining the interconnections between music performance anxiety, teaching anxiety, and the self-efficacy of preservice music teachers. Prior research has primarily highlighted a significant, inverse association between musical performance anxiety scale scores and self-efficacy beliefs related to piano performance among Turkish student teachers (Egilmez, 2015). Furthermore, only one study has delved into the relationships between music performance anxiety and teaching anxiety (Strong, 2013).

The purpose of this study was to explore the relationships among music performance anxiety, teaching anxiety, and self-efficacy among preservice music teachers in China. Four main research questions are included: (1) What are the relationships among MPA, TA, and SE? (2) What variables (gender, grade, primary instrument, secondary instrument, music learning time length, and time spent in four activities: peer teaching, practicum, internship, and private teaching) predict MPA, TA, and SE? (3) To what extent do MPA, TA, and SE predict each other? (4) Is there a difference in gender, grade, primary instruments, and secondary instruments among MPA, TA, and SE?

## Methodology

### Participants

Following approval from our affiliating universities’ institutional review boards, we recruited 246 third-and fourth-year undergraduate music education majors using purposive and snowball sampling methods. These participants were selected from seven universities located in seven areas in China: Zhejiang, Jiangsu, Hunan, Hainan, Guangdong, Anhui, and Shanghai. Out of the 246 students, 96.3% (*N* = 237) agreed to participate and completed the survey. Participants who were not identified as third or fourth year were excluded from subsequent analyses, resulting in the removal of nine participants.

The participants self-identified as 72.6% female and 27.4% male. Among them, 69.2% were third-year, while 30.8% were fourth-year. In terms of emphasis within their music studies, 43% were voice emphasis, followed by 38% in piano and 19% in other instruments. Participants also reported their secondary instruments as the piano (34.1%), voice (27%), other instruments (24.5%), choral conducting (1.3%), and none (13.1%). Most participants (32.9%) had been studying music for over 10 years, while only 5.9% had less than 3 years of music education experience.

### Measurement instruments

Instruments in this study included a consent form, demographic information, and three surveys: the Kenny Music Performance Anxiety Inventory (Kenny, 2009), the Preservice Music Teacher Self-Efficacy Scale (Prichard, 2017), and the Teaching Anxiety Scale (Parsons, 1973). In the demographic section, participants were asked to report their gender, grade level, primary instrument, secondary instrument, music learning time length, and time spent in four activities: peer teaching, practicum, internship, and private teaching. The objective was to investigate the relationships among performance anxiety, teacher self-efficacy, and teaching anxiety and assess the extent to which the first two variables could predict the third.

#### Preservice music teacher efficacy scale

Prichard’s (2017) Preservice Music Teacher Self-Efficacy Scale (PMTES) is a self-reported inventory consisting of two subscales with a total of 18 items. The first subscale, Music Teaching Efficacy Beliefs (MTE), comprises 11 items that focus on an individual’s beliefs regarding their effectiveness as a music educator. The second subscale, Classroom Management Efficacy Beliefs (CME), includes 7 items and is centered around an individual’s beliefs about their ability to manage behavioral and other non-content-area classroom situations. Both subscales demonstrated strong reliability (MTE, *α* = 0.93; CME, *α* = 0.91). To assess responses, a 6-point Likert scale ranging from 1 = strongly disagree to 6 = strongly agree was employed.

#### Kenny music performance anxiety inventory

The Kenny Music Performance Anxiety Inventory (K-MPAI) revised version (2009) comprises 8 subscales with 40 items, including proximal somatic anxiety and worry about performance (11 items, *α* = 0.91), worry/dread focused on self/other scrutiny (8 items, *α* = 0.86), depression/hopelessness (8 items, *α* = 0.85), parental empathy (4 items, *α* = 0.75), memory (2 items, *α* = 0.92), generational transmission of anxiety (3 items, α =0.72), anxious apprehension (3 items, *α* = 0.59) and biological vulnerability (1 item). Respondents would self-rate through a 7-point Likert scale from 0 to 6 (0 = strongly disagree, 6 = strongly agree). Among the 40 items, 8 items were reverse scored.

#### The teaching anxiety scale

The Teaching Anxiety Scale (TCHAS) is a self-reported inventory consisting of 25 items that was designed and established by Parsons (1973). The TCHAS is still considered the most effective way of measuring teaching anxiety for both preservice and in-service teachers. In this measure, a 5-point scale is used: 1 = never, 2 = seldom, 3 = occasionally, 4 = frequently, and 5 = often.

### Procedures

After confirming the suitability of the three inventories and obtaining permission from the instrument designers, the first author, fluent in both English and Chinese, initially translated all three instruments from English to Chinese. Following this translation, the second author, also bilingual, conducted a backward translation of the instruments from Chinese to English. Subsequently, the two authors compared the translated versions and made further adjustments to the Chinese translations. Prior to distributing the survey instrument, three undergraduate and graduate students majoring in music education took part in a pre-study to refine the translation of the three survey instruments.

Data collection was carried out using a Chinese online-based survey company, wjx.cn, a platform that collects participants’ responses, stores, and manages data, and facilitates the export of raw data, similar to Qualtrics. The website provided a template that we customized to align with the design of this study. After completing the survey design, the website automatically generated a QR code for the survey instrument. To recruit participants, the second author created an electronic flyer containing inclusion criteria and the associated QR code. Prospective participants could scan the QR code if they wished to take part in the study.

This study employed purposeful snowball sampling to recruit potential participants via WeChat, a popular social media platform in China. Recruitment assistance was provided by (1) music educators from various universities, (2) individuals known to be third- and fourth-year music education majors, and (3) parents of third- or fourth-year music education students. Upon contact, participants were invited to participate if they met the study’s criteria and expressed interest. They were also encouraged to forward the flyer to potential participants who might be interested and meet the study’s inclusion criteria.

## Results

The interitem correlations of each instrument were computed via SPSS 27, with coefficients ranging from 0.13 to 0.74 (SE), −0.30 to 0.75 (MPA), and − 0.05 to 0.65 (TA). The item-total correlations of each instrument ranged from 0.38 to 0.75 (SE), 0.00 to 0.69 (MPA) and 0.20 to 0.59 (TA), respectively. We further calculated the internal consistency of each instrument, and the results revealed that all three instruments exhibited high reliability, with Cronbach’s alpha values of 0.93 (SE), 0.93 (MPA), and 0.88 (TA), respectively (see Table 1).

**Table 1:** Descriptive statistics of the music performance anxiety, preservice music teacher self-efficacy, and teaching anxiety scales.

	Mean	SD	Skewness	Kurtosis	Interitem correlation	Internal consistency
MPA	4.05	0.74	−0.54	1.28	−0.30 to 0.75	0.93
SE	4.42	0.62	−0.19	0.75	0.13 to 0.74	0.93
TA	2.80	0.45	−0.21	0.81	−0.05 to 0.65	0.88

*N* = 237.

Due to the negative interitem correlations exhibited in music performance anxiety (MPA) and teaching anxiety (TA), we excluded items that were negatively correlated in subsequent analyses and recalculated the interitem correlation, item-total correlations, and coefficient alpha (see Table 2). The results indicated that 3 items (item 1, item 2, and item 4) in MPA and 2 items (item 1 and item 5) in TA were excluded (see Supplementary Appendix A).

**Table 2:** Descriptive statistics of recalculated music performance anxiety, preservice music teacher self-efficacy, and teaching anxiety.

	Mean	SD	Skewness	Kurtosis	Interitem correlation	Item-total correlation	Coefficient alpha
MPA	4.09	0.77	−0.56	1.26	0.02–0.81	0.17–0.76	0.93
SE	4.42	0.62	−0.19	0.75	0.13–0.74	0.38–0.75	0.93
TA	2.77	0.47	−0.26	0.79	0.74–0.61	0.28–0.61	0.88

*N* = 237.

Research Question 1: What are the relationships among MPA, TA, and SE?

Bivariate Pearson Product–Moment correlation coefficients were used to examine the relationships of music performance anxiety, preservice music teachers’ self-efficacy, and teaching anxiety. All correlations were statistically significant at the *p* < 0.01 level. The results indicated a moderately strong and positive correlation between music performance anxiety and teaching anxiety (*r = 0*.59, *p* < 0.001). However, a moderately negative correlation was found between preservice music teachers’ self-efficacy and teaching anxiety (*r* = −0.55, *p* < 0.001). Additionally, a significant negative correlation was revealed between music performance anxiety and preservice music teachers’ self-efficacy (*r* = −0.28, *p* < 0.000). In other words, results revealed that the higher the self-efficacy, the lower music performance anxiety and teaching anxiety; Simultaneously, the increased music performance anxiety was associated with an increased teaching anxiety.

In addition, we conducted partial correlation analyses to examine the relationships between (a) preservice music teachers’ self-efficacy and (b) teaching anxiety and performance anxiety, with MPA and TA being controlled, respectively. The results indicated that with MPA controlled, a significant negative correlation was found between teaching anxiety and preservice music teachers’ self-efficacy (*r* = −0.49, *p* < 0.001).

Research Question 2: What variables (gender, grade, primary instrument, secondary instrument, music learning time length, and time spent in four activities: peer teaching, practicum, internship, and private teaching) predict MPA, TA, and SE?

Research Question 3: To what extent do MPA, TA, and SE predict each other?

### Music performance anxiety

Table 3 shows the results of three stepwise multiple regression analyses. To control for Type I errors, a Bonferroni correction was applied (*α* = 0.0167 instead of *α* = 0.05). To determine the best prediction model for preservice music teachers’ music performance anxiety, we first conducted a stepwise multiple regression analysis using gender, grade, primary instrument, secondary instrument, music learning time length, time spent in four activities (peer teaching, practicum, internship, and private teaching), TA and SE as the predictors and music performance anxiety as the criterion variable. Preliminary analyses confirmed no violation of the assumptions of normality, linearity, multicollinearity, and homoscedasticity. The results of the first stepwise regression analysis reveal the best prediction model in which teaching anxiety, piano as primary instrument, and secondary instrument explained a total of 37.9% of the variance in music performance anxiety, *F*(3, 236) = 47.49. The results showed that with a one-unit increase in teaching anxiety, music performance anxiety increased by 1.57, *β* = 0.1.57, *p* < 0.001. Compared to students with other primary instruments, students who used piano as their primary instrument had an 11.75 higher music performance anxiety mean score, *β* = 11.75, *p* < 0.001. Teaching anxiety accounted for 35.1% of the variance. Primary instruments such as piano contributed an additional 1.7%.

**Table 3:** Results of stepwise regression analyses for preservice music teachers’ music performance anxiety, teaching anxiety, and self-efficacy.

Criterion variable	Predictor variables	Beta	*r*	*R*	*R^2^*	*R^2^* change
Music performance anxiety	Teaching anxiety	1.57	0.59**	0.59	0.35	0.35
	Primary piano	11.75	0.17*	0.61	0.37	0.02
Teaching anxiety	Music performance anxiety	0.18	0.59**	0.59	0.35	0.35
	Preservice music teacher’s self-efficacy	−0.38	−0.28**	0.71	0.51	0.16
	Peer teaching	−2.57	−0.11	0.72	0.52	0.01
Preservice music teacher self-efficacy	Teaching anxiety	−0.53	−0.55**	0.55	0.30	0.30
	Grade	−3.26	−0.25**	0.56	0.32	0.02

**Correlation is significant at the 0.01 level (two-tailed). *Correlation is significant at the 0.05 level (two-tailed).

### Teaching anxiety

To test which variables significantly predicted preservice music teachers’ teaching anxiety, we conducted another stepwise regression analysis with the same 11 predictors except MPA. The TA score served as the criterion variable. This stepwise multiple regression analysis revealed that music performance anxiety, self-efficacy and peer teaching were significant predictors of preservice music teachers’ teaching anxiety. A model including these three predictors was the best prediction model, accounting for 51.9% of the variance in preservice music teachers’ teaching anxiety, which was significant, *F*(3, 236) = 83.96, *p* < 0.001. The results showed that with each unit increase in music performance anxiety, teaching anxiety increased by 0.18, *β* = 0.18, *p* < 0.001, and it contributed 35.1% of the variance. With each unit increase in preservice music teachers’ self-efficacy, teaching anxiety decreased by 0.38, *β* = −0.38, *p* < 0.001, and it contributed an additional 15.5% of the variance. Teaching anxiety also decreased by 2.57 with every unit of peer teaching increase, *β* = −2.57, *p* < 0.01, and it shared 1.3% of the variance.

### Preservice music teacher self-efficacy

In the third stepwise multiple regression analyses, we regressed preservice music teachers’ self-efficacy on 11 predictors, including gender, grade, primary instrument, secondary instrument, music learning time length, time spent in four activities (peer teaching, practicum, internship, and private teaching), MPA and TA. Preservice music teachers’ self-efficacy was the criterion variable. The results indicated that teaching anxiety and grade were significant predictors, and the best model explained a total of 31% of the variance in preservice music teachers’ self-efficacy, which was significant, *F*(2,236) = 54.13. With every unit increase in teaching anxiety, teachers’ self-efficacy decreased by 0.53, *β* = −0.53, *p* < 0.001, and it contributed 29.7% of the variance. Compared to participants at other grade levels, junior participants had 3.26 points lower teacher self-efficacy, *β* = −3.26, *p* < 0.01, which contributed an additional 1.9%.

Research Question 4: Is there a difference in gender, grade, primary instruments, and secondary instruments among MPA, TA, and SE?

We computed one mixed-design MANOVA with four between-subject variables (gender, grade level, primary instrument, secondary instrument) and three within-subject variables (MPA, TA, and SE). The primary instrument encompassed three groups: voice, piano and other instruments, while the secondary instrument included five groups: voice, piano, conducting, other instruments, and no instrument. Relationship strength was determined using partial eta squared (η^2^), and an alpha level of 0.05 was set. Since the Box *M* test indicated the violation of the assumption of sphericity, the more robust Pillai’s trace was used. The results revealed that gender significantly differed for preservice music teachers’ self-efficacy *p* < 0.05, *F* (1,237) = 4.07, η^2^ = 0.02, indicating that male participants (*M* = 82.93, *SD* = 1.82) displayed a significantly higher mean score for teachers’ self-efficacy than female participants (*M* = 78.88, *SD* = 1.48). Additionally, no significant differences or significant interactions were found for the other variables (see Table 4).

**Table 4:** Results for MPA, TA and SE in gender, grade, primary instruments and secondary instruments.

Dependent variables/Independent variables	*F*	*df*	*p*
MPA			
Gender	1.29	1	NS
Grade	0.02	1	NS
Primary instrument	1.51	2	NS
Secondary instrument	1.52	4	NS
TA			
Gender	1.51	1	NS
Grade	1.05	1	NS
Primary instrument	0.78	2	NS
Secondary instrument	0.58	4	NS
SE			
Gender	4.07	1	0.045
Grade	2.19	1	NS
Primary instrument	0.84	2	NS
Secondary instrument	0.59	4	NS

## Discussion

We aimed to investigate the associations among music performance anxiety (MPA), self-efficacy (SE), and teaching anxiety (TA) among Chinese preservice music teachers. To the best of our knowledge, this study fills research gaps in the relationships among these three factors affecting preservice music teachers. Moreover, we further attempted to examine potential variables that could predict SE, MPA, and TA.

The primary result revealed a significant negative correlation between music performance anxiety and preservice music teachers’ self-efficacy, confirming that as MPA increased, self-efficacy decreased in preservice music teachers. This finding aligns with the study conducted by Dempsey and Comeau (2019), however, it contrasts with the conclusion reached by MacAfee and Comeau (2020), who found no observed relationships between music performance anxiety and self-efficacy. These differences may be attributed to variations in the research design and the target participants in these two studies. MacAfee and Comeau (2020) conducted a multiple case study over 6 weeks of intervention with five young musicians. Participants completed the Music Performance Anxiety Inventory for Adolescents (MPAI-A), Self-efficacy for Musical Performing, Performance evaluations, and Behavioral Anxiety Index in three stages (preintervention, baseline-intervention, and return to baseline). In our study, we implemented K-MPAI, PMTES, and TCHAS for 237 junior and senior preservice music teachers with diverse demographic backgrounds and various music teaching experiences. This relatively large sample size enhanced the reliability of our findings. Another potential reason for the disparities in findings might be due to the differences of the target participants. Our study focused on Chinese preservice music teachers, while MacAfee and Comeau (2020) analyzed performance anxiety and self-efficacy in Canadian adolescent young musicians. Thus, based upon discrepant research designs and diverse target participants, different studies may possess inconsistent results.

An intriguing finding is that there was an inconsistency observed between the results of the Pearson correlation analysis and the partial correlation analysis. The findings reveal that no significant correlation was found between MPA and SE through the partial correlation analysis, suggesting the presence of a confounding variable, TA. It became apparent that without controlling TA, MPA, and SE exhibited a significant correlation. One potential explanation for the inconsistency in the results could be that the role of a music teacher is multifaceted, encompassing not only teaching but also musicianship. The demonstration of musical skills is integral to the teaching process. This includes vocal demonstrations, piano accompaniment, conducting, and demonstrations on various instruments. Consequently, the factors of teaching anxiety and performance anxiety often interact in complex ways, which may influence the observed correlations.

In our study, teaching anxiety is one of the predictors of self-efficacy among preservice music teachers. In other words, preservice music teachers who experience high levels of teaching anxiety tend to have lower self-efficacy in music instruction; meanwhile, those who experience low levels of teaching anxiety tend to have higher self-efficacy in music instruction. This finding could be explained by Bandura’s theory (Bandura, 1997), as he pointed out that one’s self-efficacy beliefs were influenced by four sources of efficacy: mastery experiences, vicarious experiences, social persuasion, and emotional states. Teaching anxiety maybe related to emotional states that might influence self-efficacy. It is not surprising that the current study confirmed that teaching anxiety could predict preservice music teachers’ self-efficacy. In addition, the broader literature confirmed the current results and suggested that the increased teaching anxiety may be due to pedagogy, evaluation, classroom management, and misbehavior of students (Merç, 2015; Gorospe, 2022). These potential reasons would further affect preservice teachers’ self-efficacy.

The results of the current study indicated that no significant difference was observed between genders in music performance anxiety, which is congruent with the results of Dempsey and Comeau’s (2019) study. Nevertheless, Osborne and Kenny’s (2008) and Egilmez’s (2015) study revealed that there was a significant difference in gender and music performance anxiety. In their studies, females had higher scores on music performance anxiety than males. We assumed that one of the reasons for the lack of a specific difference in the current study could be attributed to the substantial imbalance in the male-to-female ratio in our sample. However, this imbalanced phenomenon mirrors the real-world demographic information on gender in Chinese music teacher preparation programs. In most institutions, the numbers of female students and male students in the music department are unequal: female music students are far more common than male music students, which impacted the data collection and analysis in this study.

## Implication, limitation, and future research

Results of this study imply that teacher educators should consider interventions in MPA and TA, to improve overall teacher preparedness. Interventions available include teaching practicum, mental and visual rehearsal, peer support and parents’ support, etc. (Kenny and Osborne, 2006; Prichard, 2017; Huang and Song, 2021). A limitation of this study is the small sample size and lack of gender diversity. This study recruited only 237 participants, with an unequal number of men and women. Although preservice male music teachers exhibited higher mean scores than preservice female music teachers in self-efficacy, that was not the case for music performance anxiety (MPA) and teaching anxiety (TA). In MPA and TA, no gender differences were found.

Furthermore, we propose several directions for future research. Firstly, considering the limitation of this study, it is advisable to expand the sample size to include participants with a broader range of demographic backgrounds and gender diversity. Secondly, we might consider conducting experimental research involving instructional interventions, such as field experiences and peer teaching, that could yield valuable insights into how Chinese pre-service music teachers can enhance their self-efficacy in music teaching and reduce teaching anxiety. Thirdly, we might consider a mixed methods study to explore the coping strategies of individual pre-service music teachers in dealing with teaching anxiety and music performance anxiety to gain insights on how these strategies influence their self-efficacy in teaching. Lastly, since self-efficacy is a complex and multidimensional construct (Bandura, 1986, 1997), influenced by personal traits as a variable (Topoğlu, 2014; Biasutti and Concina, 2018; Biasutti et al., 2021), we might consider exploring the relationship between personal traits and the self-efficacy of pre-service music teachers in other cultural contexts.

## Data availability statement

The raw data supporting the conclusions of this article will be made available by the authors, without undue reservation.

## Ethics statement

The studies involving humans were approved by Guangzhou University and The Pennsylvania State University's institutional review boards (IRB-STUDY00021646). The studies were conducted in accordance with the local legislation and institutional requirements. Written informed consent for participation was not required from the participants or the participants' legal guardians/next of kin because participants signed the electronic consent form.

## Author contributions

CC: Data curation, Formal analysis, Investigation, Methodology, Validation, Writing – original draft, Writing – review & editing. XX: Writing – original draft, Writing – review & editing, Methodology. YY: Formal analysis, Software, Writing – review & editing.
