# Supplementary material for: Exploring the relationships among music performance anxiety, teaching anxiety, and self-efficacy of Chinese preservice music teachers
Source: Front Psychol. 2024 Apr 12;15:1373454. doi: 10.3389/fpsyg.2024.1373454 (PMC11048473; doi:10.3389/fpsyg.2024.1373454)
Supplement: Supplementary file 1 [file Table_1.docx]

Supplementary Material

# Supplementary Table

*List of Deleted Items*

| Instrument | Items |
| --- | --- |
| MPA | Item 1. I generally feel in control of my life |
|  | Item 2. I find it easy to trust others. |
|  | Item 4. I often find it difficult to work up the energy to do things. |
| TA | Item 1. I am not confident about my improvisation ability in class. |
|  | Item 5. I am not nervous if the supervisor informs me that he/she will attend my class. |
